# Household Transmission of Community-Associated Methicillin-Resistant *Staphylococcus Aureus*

**DOI:** 10.3389/fpubh.2021.658638

**Published:** 2021-05-31

**Authors:** Feiteng Zhu, Hemu Zhuang, Shujuan Ji, Er Xu, Lingfang Di, Zhengan Wang, Shengnan Jiang, Haiping Wang, Lu Sun, Ping Shen, Yunsong Yu, Yan Chen

**Affiliations:** ^1^Department of Infectious Diseases, School of Medicine, Sir Run Run Shaw Hospital, Zhejiang University, Hangzhou, China; ^2^Key Laboratory of Microbial Technology and Bioinformatics of Zhejiang Province, Hangzhou, China; ^3^State Key Laboratory for Diagnosis and Treatment of Infectious Diseases, Collaborative Innovation Centre for Diagnosis and Treatment of Infectious Diseases, The First Affiliated Hospital of Medicine School, Zhejiang University, Hangzhou, China; ^4^Department of Hospital Epidemiology and Infection Control, School of Medicine, Sir Run Run Shaw Hospital, Zhejiang University, Hangzhou, China

**Keywords:** methicillin-resistant *Staphylococcus aureus*, whole-genome sequencing, households transmission, pets, colonization

## Abstract

Currently, the mechanism of community-associated methicillin-resistant *Staphylococcus aureus* (CA-MRSA) transmission mechanism is unclear; however, it must be considered in conjunction with asymptomatic *S. aureus* strains colonization dynamics. This epidemiological study aimed to determine the role of the household in CA-MRSA transmission in China. Five patients with culture-confirmed CA-MRSA infection and five control patients were recruited from the Sir Run Run Shaw Hospital in Zhejiang, China, between December 2019 and January 2020. The household members of the patients, their pets, and environmental surfaces were sampled and screened for MRSA colonization. Mass spectrometry identification and antimicrobial susceptibility testing were performed on the MRSA isolates. Whole-genome sequencing and core genome multilocus sequence typing (cgMLST) were performed to determine the origin and transmission of the MRSA isolates in the households. Overall, 14 *S. aureus*-positive specimens (14.1%, 14/99) were obtained from the five households of patients with CA-MRSA infections, of which 12 (85.7%) were MRSA. The overall positivity of MRSA was 12.1% (12/99) among the samples from the CA-MRSA households, while no MRSA isolates were detected in the five control households. Most MRSA isolates belonged to epidemic CA-MRSA clones, such as ST59 (15/35, 42.9%) and ST508 (15/35, 42.9%). The cgMLST results confirmed that MRSA was transmitted among patients, contacts, and pets in the households and was present on environmental surfaces in the CA-MRSA patients' households. In conclusion, the study revealed that the home environment was an important MRSA reservoir. Therefore, focusing on MRSA decolonization in patients alone is not sufficient for infection control of CA-MRSA.

## Introduction

*Staphylococcus aureus* is the main cause of infections in hospitals and communities and contributes significantly to the healthcare burden (1). *S. aureus* can cause various infections, from asymptomatic to invasive infections and from mild skin and soft tissue infections to life-threatening bacteremia. Since the mid-1990s, an increase in methicillin-resistant *S. aureus* (MRSA) infections has been reported among populations without exposure risk within the healthcare system, caused by community-associated MRSA (CA-MRSA) strains (2). However, the CA-MRSA dissemination and transmission mechanisms remain unclear.

Individuals who are asymptomatic carriers of *S. aureus* are the primary natural reservoir (3). Studies of CA-MRSA among family contacts of patients with CA-MRSA infection have found MRSA colonization rates of 8.7–37% (4). Unrecognized colonization of household contacts and contamination of the home environment may maintain colonization among individuals with MRSA. In some case reports, individuals who underwent appropriate antibiotic treatment for several months were cured only after disinfecting contaminated household surfaces (5, 6). In addition, methicillin-sensitive *S. aureus* (MSSA) and MRSA have been recovered from environmental samples collected in homes in which none of the inhabitants had signs of apparent infection (7, 8). There are still many unanswered questions about CA-MRSA colonization in the family environment. Some specific MRSA clones, such as ST-59 and ST-508, are prevalent in China. However, the specific transmission mechanism of CA-MRSA is unclear (9).

Thus, we conducted a pilot epidemiological study of the households of patients with CA-MRSA infection in China. We aimed to determine the potential role of other household members and the household environment in CA-MRSA transmission.

## Materials and Methods

### Participant Recruitment

Five patients diagnosed with CA-MRSA infection, including three with skin and soft tissue infections, one with septic arthritis, and one with mastoiditis, were enrolled in the study between December 2019 and January 2020. According to the United States Centers for Disease Control and Prevention definition, CA-MRSA infection is defined as any MRSA infection diagnosed in an outpatient or within 48 h of hospital admission in the absence of healthcare-associated MRSA risk factors. MRSA risk factors include hemodialysis, surgery, residence in a long-term care facility, hospitalization during the previous year, presence of an indwelling catheter or percutaneous device at culture time, or previous MRSA isolation from the patient (2). Five healthy subjects without *Staphylococcus* infection and their households were enrolled as the control group for comparison. These healthy subjects were all adult volunteers who lived in Zhejiang province without the following exclusion criteria: a history of *S. aureus* infection or colonization, chronic diseases and residence in a long-term care facility or hospitalization during the previous year, and medical workers. The five patients' households were labeled as A, B, C, D, and E, and the five control households were labeled F, G, H, I, and J.

### Data and Specimen Collection

**Figure 1:**
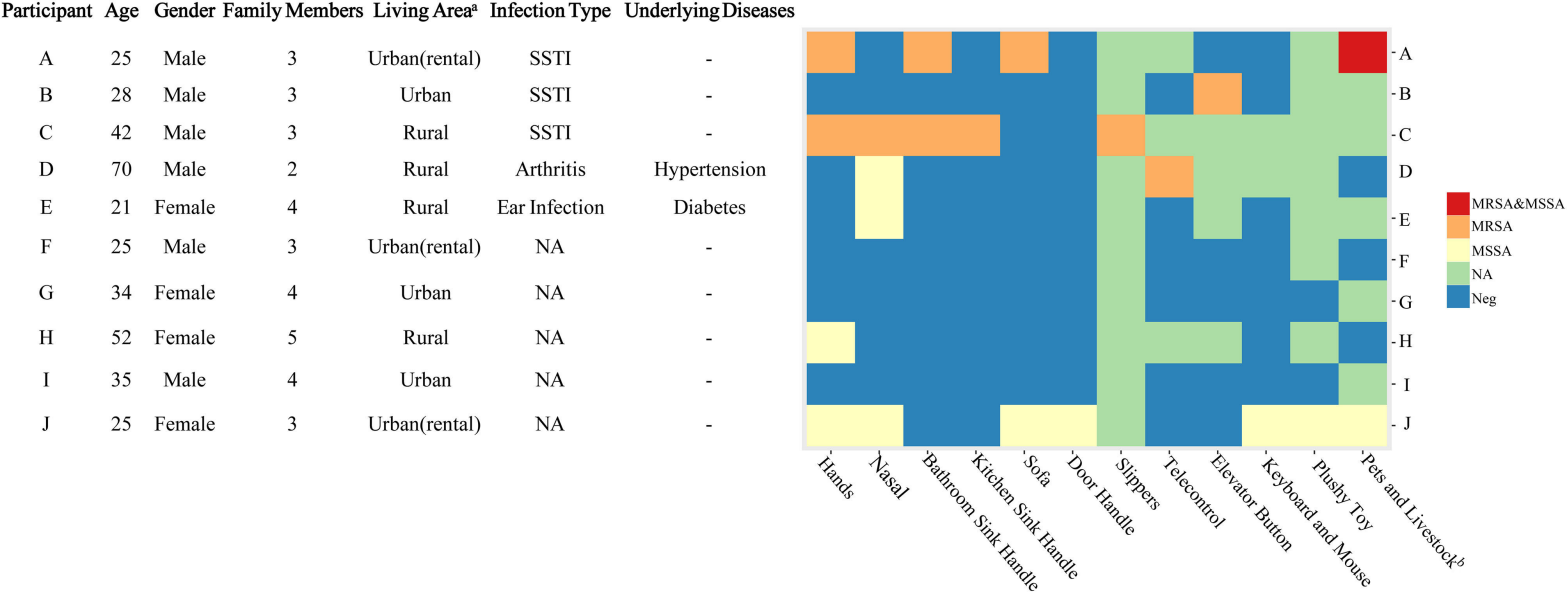
Characteristics of the participants and the methicillin-resistant *Staphylococcus aureus* isolates in their households. A, B, C, D, and E were the patients with CA-MRSA infection, and F, G, H, I, and J were the healthy subjects. CA, community-acquired; MRSA, methicillin-resistant *Staphylococcus aureus*; MSSA, methicillin-susceptible *Staphylococcus aureus*; NA, not available; Neg, negative; SSTI, skin and soft tissue infection. ^a^Urban areas are densely populated with populations of ≥800,000. ^b^Dogs, cats, and chickens were included.

One visit was conducted at the home of each case patient. Samples were collected from four sources: the home environment, household members, pets, and poultry (10). Environmental surfaces presumed to be frequently touched (handles of doors, bathroom basin and kitchen sink, sofas, and computer keyboard and mouse) and used by most household members or presumed to play a role in transmission were sampled. We also collected samples from the nostrils of each family member and hairs on the backs of pets, such as dogs and cats, and livestock (Figure 1).

Samples were obtained from each environmental surface using a pre-wet sponge bar with Neutralizing Buffer (3M, Saint Paul, MN) using standard procedures (10). All patients and healthy subjects were required to refrain from any additional cleaning measures before sampling.

### Culture, Identification, and Antimicrobial Susceptibility Testing

The samples were transported to the laboratory immediately after sampling, and each sample was inoculated into 2 mL of trypsin soy broth and incubated at 37°C overnight. The next day, 50 μL of the medium was streaked on CHROMagar Staph aureus plates (CHROMagar, Paris, France) and incubated at 37°C for 24 h. On the second day, we selected up to three pink or mauve colonies per sample and incubated them at 37°C for 24 h for further identification. Identification was performed based on mass spectrometry analysis using a microTyper MS (Skyray, Jiangsu, China) analyzer. Antimicrobial susceptibility testing was performed using agar dilution, and a D-zone test was performed according to the 2020 Clinical and Laboratory Standards Institute (CLSI) guidelines (cefoxitin, ciprofloxacin, levofloxacin, moxifloxacin, gentamicin, tetracycline, linezolid, vancomycin, trimethoprim-sulfamethoxazole, daptomycin, rifampicin, erythromycin, and clindamycin.) and the European Committee on Antimicrobial Susceptibility Testing (EUCAST) (fosfomycin) (11). *S. aureus* ATCC 29213 was used as the control strain for antimicrobial susceptibility testing (12). All the isolates, MSSA and MRSA identified from the CHROMagar plates, were stored at −80°C for further analyses.

### Whole-Genome Sequencing and Core Genome Multilocus Sequence Typing

Genomic DNA was extracted from *S. aureus* cultures using the QIAamp DNA Mini Kit (QIAGEN, Hilden, Germany) following the manufacturer's instructions. All MRSA isolates recovered from the index patients and households were sequenced on an Illumina HiSeq X-10 platform using the 2 × 150-base-pair paired-end mode (Illumina, San Diego, CA) (11). We selected one isolate from each MSSA-positive sample for sequencing. The derived short reads were assembled into contigs using CLC Genomics Workbench software (version 12.0.0; CLC bio, Aarhus, Denmark). Genome assemblies were imported into SeqSphere+ software (version 6.0.0; Ridom) as FASTA files for multilocus sequence typing (MLST), core genome multilocus sequence typing (cgMLST) analysis, and staphylococcal protein A typing. The thresholds for interpreting clonality with cgMLST were as follows: ≤8 allelic differences were considered related, 9–29 allelic differences were considered possibly related, and ≥30 allelic differences were considered unrelated (13). For each household sample, *S. aureus* isolates with identical sequence types (ST) were considered to be redundant isolates.

### Statistical Analysis

Fisher's exact test was used to determine the significance of differences in the MRSA isolation rates between the households of the patients with CA-MRSA and the control households. *P*-values of <0.05 were considered statistically significant.

### Ethics

This study was approved by the local ethics committees of Sir Run Run Shaw Hospital (approval no. 20191201-1). Written informed consent was obtained from all participants or from a guardian if the participant was a minor.

## Results

### Study Participants

The mean age of the five CA-MRSA case-patients was 37 years (range: 21–70 years), and four out of five were male (Figure 1). The average time from the day of MRSA isolation to study enrolment was 19 days (range: 6–38 days). The average distance between the patients' houses and the Sir Run Run Shaw Hospital was 43 km (range: 6–88 km). Two patients lived in urban areas (>800,000 individuals in the census), while the other three lived in rural areas. Patient A had two cats and patient D had one dog in their households. The characteristics of the households of the control subjects are shown in Figure 1.

### Prevalence of MRSA in the Household Contacts, Environment, and Pets

A total of 99 and 104 samples were collected from the households of the five CA-MRSA infection patients and healthy subjects, respectively. Up to three colonies were taken from each sample for identification and susceptibility testing as described in the methods. In total, 35 *S. aureus* isolates, including 32 MRSA and three MSSA, were identified from the samples of CA-MRSA patients' households, whereas only nine *S. aureus* isolates were identified from the samples of the healthy control households. None of the nine *S. aureus* isolates from the control households were MRSA strains. Thirty-five *S. aureus* isolates from patient households (32 MRSA and three MSSA), five MRSA isolates from index patients, and nine MSSA isolates from healthy control households were sequenced and analyzed (Supplementary Table 1). After removing the redundant *S. aureus* isolates, the total positive rate of *S. aureus* samples was 14.1% (14/99) in the case households and 8.7% (9/104) in the control households (*P* = 0.27). Of the 14 *S. aureus*-positive samples from the case households, 12 (85.7%) were MRSA strains (Table 1).

**Table 1:** Prevalence of *Staphylococcus aureus* on domestic environmental surfaces, contacts, and pets and poultry.

	**Patient households**				**Health households**				***P*-value (SA and non-SA)**
	***N***	**No. (%) of isolates**			***N***	**No. (%) of isolates**			
		**SA**	**MRSA**	**MSSA**		**SA**	**MRSA**	**MSSA**	
Household surfaces^a^	99	14 (14)	12 (12)	3 (3)	104	9 (9)	0 (0)	9 (9)	0.27
Living room	14	2 (14)	2 (14)	0 (0)	17	3 (18)	0 (0)	3 (18)	1.00
Computer keyboard and mouse	3	0 (0)	0 (0)	0 (0)	5	1 (20)	0 (0)	1 (20)	··
TV remote control	5	1 (20)	1 (20)	0 (0)	4	0 (0)	0 (0)	0 (0)	··
Sofa	5	1 (20)	1 (20)	0 (0)	5	1 (20)	0 (0)	1 (20)	··
Other items	1	0 (0)	0 (0)	0 (0)	3	1 (33)	0 (0)	1 (33)	··
Bathroom	20	2 (10)	2 (10)	0 (0)	23	0 (0)	0 (0)	0 (0)	0.210
Door handle	6	0 (0)	0 (0)	0(0)	5	0 (0)	0 (0)	0 (0)	··
Sink faucet	6	2 (33)	2 (33)	0 (0)	6	0(0)	0 (0)	0 (0)	··
Toilet seat, comb, and washing machine	8	0 (0)	0 (0)	0 (0)	11	0 (0)	0 (0)	0 (0)	··
Other items	0	0 (0)	0 (0)	0 (0)	1	0 (0)	0 (0)	0 (0)	··
Kitchen^b^	14	1 (7)	1 (7)	0 (0)	16	0 (0)	0 (0)	0 (0)	0.467
Bedroom^b^	10	0 (0)	0 (0)	0 (0)	1	0 (0)	0 (0)	0 (0)	.
Special items	16	2 (13)	2 (13)	0	21	1 (5)	0 (0)	1 (5)	0.568
Elevator button	2	1 (50)	1 (50)	0 (0)	1	0 (0)	0 (0)	0 (0)	··
Door handle and doorbell phone	6	0 (0)	0 (0)	0 (0)	10	1 (14)	0 (0)	1 (14)	··
Other items	8	1 (13)^c^	1 (13)	0 (0)	10	0 (0)	0 (0)	0 (0)	··
Contact	20	5 (25)	3 (15)	2 (10)	22	4 (18)	0 (0)	4 (18)	0.14
Hands of family members	8	2 (25)	2 (25)	0 (0)	11	2 (18)	0 (0)	2 (18)	··
Noses of family members	11	3 (27)	1 (9)	2 (18)	11	2 (18)	0 (0)	2 (18)	··
Ears of family members	1	0 (0)	0 (0)	0 (0)	0	0 (0)	0 (0)	0 (0)	··
Pets and livestock	5	2 (40)	2 (40)	1 (20)	4	1 (25)	0 (0)	1 (25)	1.00
Cats^d^	2	2 (100)	2 (100)	1 (50)	3	1 (33)	0 (0)	1 (33)	··
Dogs	1	0 (0)	0 (0)	0 (0)	1	0 (0)	0 (0)	0 (0)	··
Livestock	2	0 (0)	0 (0)	0 (0)	0	0 (0)	0 (0)	0 (0)	··

*SA, Staphylococcus aureus; MRSA, methicillin-resistant Staphylococcus aureus; MSSA, methicillin-susceptible S. aureus; ··, not applicable*.

a *We sampled the environmental surfaces, contacts, and pets and livestock present during the visit*.

b *The kitchen included other items such as countertops, cabinet handles, sink faucets, and refrigerator door handles, and the bedroom included light switches and door handles*.

c *Patient C's slippers were positive for MRSA and were classified in other items in the special item category*.

d *One cat was positive for both MRSA and MSSA*.

Among the five CA-MRSA patients' households, four had MRSA-contaminated environments, with a median number of two positive environmental samples (range: 0–5) per household. In the households of patients A, C, and D, MRSA was isolated from the handles of the bathroom basin, handles of kitchen sinks, slippers, sofa, and television remote control. However, from patient B's household, the only MRSA-positive environmental sample was obtained from the elevator button outside the patient's apartment. In patient E's household, none of the environmental surface samples tested positive for MRSA. In the control households, only MSSA isolates were found in the environmental samples from participant J's and H's households (Figure 1). We identified MRSA carriage in two cats of patient A, and one of the cats carried both MRSA and MSSA. No MRSA was isolated from the sample taken from patient D's dog.

### Antimicrobial Susceptibility Testing and Molecular Typing

The minimum inhibitory antibiotic concentrations of 12 MRSA isolates from households and five MRSA isolates from patients are shown in Table 2. Fosfomycin results were interpreted in accordance with the EUCAST while others were according to the CLSI. As can be seen from Table 2, these strains were all resistant to cefoxitin, and none were resistant to gentamicin, linezolid, vancomycin, trimethoprim-sulfamethoxazole, daptomycin, or rifampicin. Only two isolates (Patient D and his television remote control) showed intermediate to ciprofloxacin, levofloxacin, moxifloxacin. Additionally, one isolate (Patient B) was resistant to tetracycline. As for fosfomycin, 94.1% (16/17) of strains were susceptible. Moreover, 70.6% (12/17) of the MRSA isolates were resistant to erythromycin, while 29.4% (5/17) were intermediate. According to the D-test method, 17.6% (3/17) of these strains showed inducible clindamycin resistance, whereas constitutive clindamycin resistance was detected in 47.1% (8/17) of all MRSA strains.

**Table 2:** Minimum inhibitory antibiotic concentrations of MRSA isolates from patients and their households.

	**Specimen source**	**ST**	**FOX**	**CIP**	**LEV**	**MXF**	**GEN**	**TET**	**LNZ**	**VAN**	**FOS**	**SMZ**	**DAP**	**RIF**	**ERY**	**CLI**
A-MRSA1	Patient A	59	8	0.5	0.25	0.03	0.5	0.5	0.5	1	2	0.03/0.59	0.5	0.002	>256	64
A-MRSA2	Bathroom sink faucet	59	8	0.5	0.25	0.03	0.5	0.5	0.5	1	2	0.03/0.59	0.5	0.002	>256	64
A-MRSA5	Cat-A	59	8	0.5	0.25	0.03	0.5	0.5	0.5	1	2	0.03/0.59	0.5	0.002	>256	64
A-MRSA8	Cat-B	59	8	0.5	0.5	0.03	0.5	0.5	0.5	1	32	0.03/0.59	0.5	0.002	>256	64
A-MRSA9	Sofa	59	8	0.5	0.13	0.06	0.5	0.5	0.5	1	1	0.03/0.59	0.5	0.002	>256	64
A-MRSA11	Hands of patient A's girlfriend	59	16	0.5	0.25	0.03	0.5	0.5	0.5	1	1	0.03/0.59	0.5	0.002	>256	64
B-MRSA1	Patient B	59	8	0.5	0.25	0.03	0.5	32	0.5	1	1	0.03/0.59	0.5	0.002	>256	64
B-MRSA2	Elevator button	59	32	0.5	0.5	0.5	0.5	0.5	0.5	1	4	0.03/0.59	0.5	0.002	>256	0.13^a^
C-MRSA1	Patient C	508	16	0.25	0.13	0.06	0.5	0.5	0.5	1	2	0.03/0.59	0.5	0.002	1	0.13
C-MRSA2	Bathroom sink faucet	508	16	0.25	0.13	0.06	0.5	0.5	0.5	2	2	0.03/0.59	0.5	0.002	1	0.13
C-MRSA5	Kitchen sink faucet	508	16	0.25	0.13	0.06	0.5	0.5	0.5	2	2	0.03/0.59	0.5	0.002	>256	0.25^a^
C-MRSA8	Slippers of patient C	508	16	0.25	0.13	0.03	0.5	0.5	0.5	2	2	0.03/0.59	0.5	0.002	1	0.25
C-MRSA11	Hands of patient C's son	508	16	0.25	0.13	0.06	0.5	0.5	1	2	2	0.03/0.59	0.5	0.002	>256	0.13
C-MRSA14	Nose of patient C's son	508	16	0.25	0.13	0.03	0.5	0.5	1	2	2	0.03/0.59	0.5	0.002	>256	0.25^a^
D-MRSA1	Patient D	398	8	2	2	1	0.5	0.5	1	1	4	0.03/0.59	0.25	0.002	1	0.25
D-MRSA2	Television remote control	59	32	2	2	1	0.5	0.5	1	1	>128	0.03/0.59	0.5	0.002	>256	64
E-MRSA1	Patient E	398	8	0.5	0.5	0.5	0.5	1	1	1	1	0.25/4.75	0.25	0.002	1	0.25

*MRSA, methicillin-resistant Staphylococcus aureus; ST, sequence type; FOX, cefoxitin; CIP, ciprofloxacin; LEV, levofloxacin; MXF, moxifloxacin; GEN, gentamycin; TET, tetracycline; LNZ, linezolid; VAN, vancomycin; FOS, fosfomycin; SMZ, trimethoprim-sulfamethoxazole; DAP, daptomycin; RIF, rifampicin; ERY, erythromycin; CLI, clindamycin*.

a *D-zone test was positive*.

**Figure 2:**
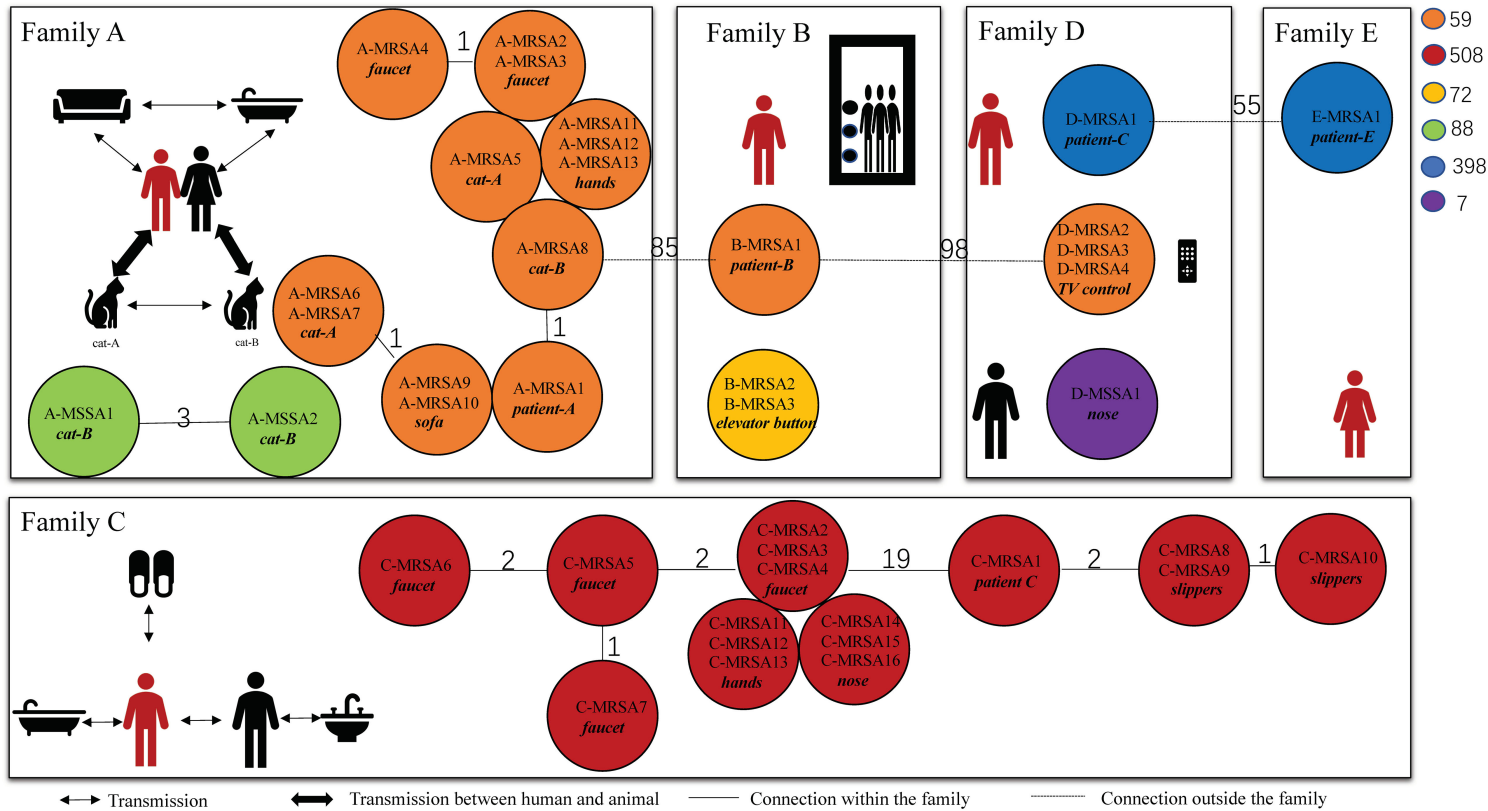
Transmission pattern suggested by core genome multilocus sequence typing of methicillin-resistant *Staphylococcus aureus*. These isolates were collected from the five patients and their households. The persons in red are patients with CA-MRSA infection, and the ones in black are family members. The different-colored circles represent different ST-type CA-MRSA strains. The number on the line between the circles of different colors represents the number of core allele differences between the isolates. The source of specific specimens is shown in Table 3. CA, community acquired; MRSA, methicillin-resistant *Staphylococcus aureus*; ST, sequence type; MSSA, methicillin-susceptible *Staphylococcus aureus*.

To determine the MRSA transmission pattern, we performed whole-genome sequencing and molecular typing of the *S. aureus* isolates recovered from the patients and their households. We found that half of the MRSA isolates were predominantly CA-MRSA clones, including ST59 and ST72 (14). The ST of the MRSA clones from patients A and C matched that of the positive household samples. Patient A was infected with MRSA ST59, and the MRSA isolates from the handles of his sink handles of bathroom, sofa, and cats had the identical ST. According to the cgMLST analysis, the MRSA isolates from patient A and his household were closely related with no allelic difference or only one allelic difference from each other. Three allelic differences between two MSSA isolates from patient A's cat were identified (Figure 2). According to the molecular typing results, the two MSSA strains were not related to the isolated MRSA strain. Similarly, the MRSA isolate ST from patient C was ST508 which was identical to the ST of the MRSA isolates from the corresponding environmental surfaces and contacts. The cgMLST results showed that the number of allele differences among these isolates varied from 1 to 19, indicating that they were related (Figure 2). The results of SCC*mec* typing are shown in Supplementary Table 2.

In patients B and D, the STs of the MRSA isolates recovered from the household environmental samples did not match those of the MRSA isolates from the patients. The MRSA isolates from patient B were ST59; however, the MRSA isolates recovered from the elevator button of his apartment were ST72. ST398 MRSA isolates were identified in patients D and E; however, ST59 MRSA on the television remote control was the only ST identified in the environmental samples from patient D. The cgMLST results confirmed that there was no relationship between the MRSA isolates from patients B and D and the isolates from their household samples. In addition, no MRSA isolates were found in the environmental samples from patient E's household.

**Table 3:** Specific sources of specimens in Figure 2.

**Source of the specimen**	**Specimen number**
Patient A	A-MRSA1
Bathroom sink faucet	A-MRSA2, A-MRSA3, A-MRSA4
Cat-A	A-MRSA5, A-MRSA6, A-MRSA7
Cat-B	A-MRSA8, A-MSSA1, A-MSSA2
Sofa	A-MRSA9, A-MRSA10
Hands of patient A's girlfriend	A-MRSA11, A-MRSA12, A-MRSA13
Patient B	B-MRSA1
Elevator button	B-MRSA2, B-MRSA3
Patient C	C-MRSA1
Bathroom sink faucet	C-MRSA2, C-MRSA3, C-MRSA4
Kitchen sink faucet	C-MRSA5, C-MRSA6, C-MRSA7
Slippers of patient C	C-MRSA8, C-MRSA9, C-MRSA10
Hands of patient C's son	C-MRSA11, C-MRSA12, C-MRSA13
Nose of patient C's son	C-MRSA14, C-MRSA15, C-MRSA16
Patient D	D-MRSA1
Television remote control	D-MRSA2, D-MRSA3, D-MRSA4
Nose of patient D's son	D-MSSA1
Patient E	E-MRSA1

*MRSA, methicillin-resistant Staphylococcus aureus; MSSA, methicillin-susceptible S. aureus*.

## Discussion

The household environment is considered an important reservoir of MRSA (15). In our study, environmental MRSA contamination was found in four of the five households of patients with CA-MRSA infections. In contrast, in healthy subjects, no MRSA was identified in their households. Our data demonstrated that the household environment plays an important role in CA-MRSA infection. It has been reported that epidemic clones tend to “ping-pong” among family members, leading to a high incidence of repeated infections (16). In a cross-sectional study by Fritz et al., MRSA was detected on environmental surfaces in 46% of households. The bed linens, television remote controls, and refrigerator door handles were most commonly colonized (17). In another study by Shahbazian et al., which included 95 homes of patients diagnosed with CA-MRSA infection, MRSA was recovered from 68% (65/95) of the homes at baseline (18). MRSA prevalence in the household environment in the present study was relatively higher than that reported in previous studies, which might be explained by the limited number of cases included in our study.

A previous study found animals to be an important reservoir of MRSA (19). Although no MRSA isolates were identified from livestock in our study, Bi et al. reported that CA-MRSA isolates with a common genotype could be isolated from humans and pigs, suggesting that human-to-pig transmission of CA-MRSA could occur (20). In our study, two cats belonging to patient A and one cat belonging to healthy subject J were colonized by *S. aureus*. MRSA isolates belonging to the epidemic CA-MRSA clone were identified from two cats recognized as the infection source according to the cgMLST findings. Our results indicated that pets can be reservoirs and play a role in *S. aureus* and MRSA transmission. In the HOME study conducted by Mork et al. in the United States, 44% (68/154) of pets sampled were colonized with *S. aureus* and 29% with MRSA; the molecular typing results showed that the pets were often transmission recipients (9). In our study, MRSA was found in two of the three pets tested, which is a higher prevalence than in other studies.

In addition to the traditional molecular type, we applied cgMLST based on whole-genome sequencing, which provides improved resolution, to trace the MRSA strains' transmission. The MRSA isolates from households were predominantly CA-MRSA clones, such as ST59, ST72, and ST508 (single-locus variants of CA-MRSA clone ST45). Specifically, two patients were infected with MRSA ST59, the predominant clone in China and other Asian countries (21). The cgMLST results also confirmed the relationships among the patients' strains, contacts, environmental surfaces, and pets. In recent years, MRSA ST59 has been recognized as an epidemic lineage in Asia which accounts for 56% of pediatric CA-MRSA infections in Taiwan (22). Moreover, it has been associated with an increasing proportion of CA-MRSA infections in mainland China (23). Pang et al. provided evidence for cross-country transmission potential of MRSA ST59 through the food chain (24). Our data demonstrated that environmental contamination and pets also contribute to the success of MRSA ST59 in China. MRSA ST398, another important lineage confirmed as a cause of livestock-associated MRSA in Asia, Australia, and the Americas, was identified in two patients in this study. Although no MRSA ST398 was recovered from the corresponding households, we could not rule out the possibility of MRSA ST398 contamination in the household because of the limited number of samples collected during a single visit. Previous studies have shown that this new CA-MRSA ST has the potential for serious and fatal infections and should be monitored for its potential spread. Environmental decontamination should be considered as a strategy to prevent the future spread of MRSA ST398.

In our study, MRSA colonization was detected in pets and household environments; thus, decolonization is important. Research about the decolonization of MRSA has been published. Ho et al. revealed that decolonization may be more likely to succeed because most CA-MRSA carriers are otherwise healthy and do not have any indwelling medical equipment that could be used as permanent settlements (25). As for methods of decolonization, Hogan et al. demonstrated a 5-day intervention that consists of intranasal mupirocin application and dilute bleach water baths could reduce MRSA colonization in the months following the one-time visit; however, this effect waned over time (26).

Our study has several limitations. Because it is a pilot study, only five patients were enrolled. A relatively small number of samples were collected from each patient's household, limiting our statistical analysis of the epidemiology of MRSA transmission and risk factors of MRSA infection. However, because epidemiological surveys of MRSA in households are lacking in China, this study provides important preliminary evidence to explain the transmission mechanism of MRSA in Chinese communities. Second, a one-time visit is insufficient to obtain a comprehensive survey of the household environment of patients with MRSA infection. However, the isolates and genome data obtained in this pilot study will help establish a household MRSA database as a basis for further research.

## Conclusions

In conclusion, this study confirmed that CA-MRSA is transmitted in the home environments of patients with CA-MRSA. Household environmental decontamination should be considered as a strategy to prevent CA-MRSA from spreading, particularly among households where an infection has occurred.

## Data Availability Statement

The original contributions presented in the study are publicly available. This data can be found here: https://bigd.big.ac.cn/bioproject/browse/PRJCA004387.

## Ethics Statement

The studies involving human participants were reviewed and approved by local ethics committees of Sir Run Run Shaw Hospital. The patients/participants provided their written informed consent to participate in this study.

## Author Contributions

YC, YY, and FZ designed the study. FZ, HZ, and ShuJ drafted the article. FZ and HZ obtained the data. EX, LD, ZW, SheJ, PS, and HW did the experiments. LS, YY, and YC revised the article. All authors have approved the final article.

## Conflict of Interest

The authors declare that the research was conducted in the absence of any commercial or financial relationships that could be construed as a potential conflict of interest.
