# Supplementary material for: Household Transmission of Community-Associated Methicillin-Resistant Staphylococcus Aureus
Source: Front Public Health. 2021 May 31;9:658638. doi: 10.3389/fpubh.2021.658638 (PMC8200482; doi:10.3389/fpubh.2021.658638)
Supplement: Supplementary file 1 [file Table_1.DOCX]

Supplementary Material

# Supplementary Tables

## Supplementary Table 1. The minimum inhibitory antibiotic concentrations of MRSA and MSSA strains sampled from patients, healthy controls, and their households.

|  | Specimen source | FOX | CIP | LEV | MXF | GEN | TET | LNZ | VAN | FOS | | SMZ | DAP | RIF | ERY | CLI |
| --- | --- | --- | --- | --- | --- | --- | --- | --- | --- | --- | --- | --- | --- | --- | --- | --- |
| A-MRSA1 | Patient A | 8 | 0.5 | 0.25 | 0.03 | 0.5 | 0.5 | 0.5 | 1 | 2 | 0.03/0.59 | | 0.5 | 0.002 | >256 | 64 |
| A-MRSA2 | Bathroom sink faucet | 8 | 0.5 | 0.25 | 0.03 | 0.5 | 0.5 | 0.5 | 1 | 2 | 0.03/0.59 | | 0.5 | 0.002 | >256 | 64 |
| A-MRSA3 | Bathroom sink faucet | 8 | 0.5 | 0.25 | 0.03 | 0.5 | 0.5 | 0.5 | 1 | 1 | 0.03/0.59 | | 0.5 | 0.002 | >256 | 64 |
| A-MRSA4 | Bathroom sink faucet | 8 | 0.5 | 0.25 | 0.03 | 0.5 | 0.5 | 0.5 | 1 | 1 | 0.03/0.59 | | 0.5 | 0.002 | >256 | 64 |
| A-MRSA5 | Cat-A | 8 | 0.5 | 0.25 | 0.03 | 0.5 | 0.5 | 0.5 | 1 | 2 | 0.03/0.59 | | 0.5 | 0.002 | >256 | 64 |
| A-MRSA6 | Cat-A | 8 | 0.5 | 0.25 | 0.03 | 0.5 | 0.5 | 0.5 | 1 | 1 | 0.03/0.59 | | 0.5 | 0.002 | >256 | 64 |
| A-MRSA7 | Cat-A | 8 | 0.5 | 0.25 | 0.03 | 0.5 | 0.5 | 0.5 | 1 | 2 | 0.03/0.59 | | 0.5 | 0.002 | >256 | 64 |
| A-MRSA8 | Cat-B | 8 | 0.5 | 0.5 | 0.03 | 0.5 | 0.5 | 0.5 | 1 | 32 | 0.03/0.59 | | 0.5 | 0.002 | >256 | 64 |
| A-MSSA1 | Cat-B | 4 | 0.5 | 0.25 | 0.5 | 0.5 | 32 | 0.5 | 1 | 1 | 0.03/0.59 | | 0.5 | 0.002 | 1 | 0.25 |
| A-MSSA2 | Cat-B | 4 | 0.5 | 0.25 | 0.5 | 0.5 | 32 | 0.5 | 1 | 1 | 0.03/0.59 | | 0.5 | 0.002 | 1 | 0.25 |
| A-MRSA9 | Sofa | 8 | 0.5 | 0.13 | 0.06 | 0.5 | 0.5 | 0.5 | 1 | 1 | 0.03/0.59 | | 0.5 | 0.002 | >256 | 64 |
| A-MRSA10 | Sofa | 8 | 0.5 | 0.13 | 0.5 | 0.5 | 0.5 | 0.5 | 1 | 32 | 0.03/0.59 | | 0.5 | 0.002 | >256 | 64 |
| A-MRSA11 | Hands of patient A’s girlfriend | 16 | 0.5 | 0.25 | 0.03 | 0.5 | 0.5 | 0.5 | 1 | 1 | 0.03/0.59 | | 0.5 | 0.002 | >256 | 64 |
| A-MRSA12 | Hands of patient A’s girlfriend | 8 | 0.5 | 0.25 | 0.03 | 0.5 | 0.5 | 0.5 | 1 | 1 | 0.03/0.59 | | 0.5 | 0.002 | >256 | 64 |
| A-MRSA13 | Hands of patient A’s girlfriend | 8 | 0.5 | 0.25 | 0.03 | 0.5 | 0.5 | 0.5 | 1 | 1 | 0.03/0.59 | | 0.5 | 0.002 | >256 | 64 |
| B-MRSA1 | Patient B | 8 | 0.5 | 0.25 | 0.03 | 0.5 | 32 | 0.5 | 1 | 1 | 0.03/0.59 | | 0.5 | 0.002 | >256 | 64 |
| B-MRSA2 | Elevator button | 32 | 0.5 | 0.5 | 0.5 | 0.5 | 0.5 | 0.5 | 1 | 4 | 0.03/0.59 | | 0.5 | 0.002 | >256 | 0.13 |
| B-MRSA3 | Elevator button | 32 | 0.5 | 0.5 | 0.5 | 0.5 | 0.5 | 0.5 | 1 | 4 | 0.03/0.59 | | 0.5 | 0.002 | >256 | 0.13 |
| C-MRSA1 | Patient C | 16 | 0.25 | 0.13 | 0.06 | 0.5 | 0.5 | 0.5 | 1 | 2 | 0.03/0.59 | | 0.5 | 0.002 | 1 | 0.13 |
| C-MRSA2 | Bathroom sink faucet | 16 | 0.25 | 0.13 | 0.06 | 0.5 | 0.5 | 0.5 | 2 | 2 | 0.03/0.59 | | 0.5 | 0.002 | 1 | 0.13 |
| C-MRSA3 | Bathroom sink faucet | 16 | 0.25 | 0.13 | 0.06 | 0.5 | 0.5 | 0.5 | 2 | 2 | 0.03/0.59 | | 0.5 | 0.002 | 1 | 0.25 |
| C-MRSA4 | Bathroom sink faucet | 16 | 0.25 | 0.13 | 0.06 | 0.5 | 0.5 | 0.5 | 2 | 2 | 0.03/0.59 | | 0.5 | 0.002 | 1 | 0.25 |
| C-MRSA5 | Kitchen sink faucet | 16 | 0.25 | 0.13 | 0.06 | 0.5 | 0.5 | 0.5 | 2 | 2 | 0.03/0.59 | | 0.5 | 0.002 | >256 | 0.25 |
| C-MRSA6 | Kitchen sink faucet | 16 | 0.25 | 0.13 | 0.03 | 0.5 | 0.5 | 0.5 | 2 | 2 | 0.03/0.59 | | 0.5 | 0.002 | >256 | 0.25 |
| C-MRSA7 | Kitchen sink faucet | 16 | 0.25 | 0.13 | 0.03 | 1 | 0.5 | 0.5 | 1 | 2 | 0.03/0.59 | | 0.5 | 0.002 | >256 | 0.25 |
| C-MRSA8 | Slippers of patient C | 16 | 0.25 | 0.13 | 0.03 | 0.5 | 0.5 | 0.5 | 2 | 2 | 0.03/0.59 | | 0.5 | 0.002 | 1 | 0.25 |
| C-MRSA9 | Slippers of patient C | 16 | 0.25 | 0.13 | 0.03 | 0.5 | 0.5 | 0.5 | 1 | 2 | 0.03/0.59 | | 0.5 | 0.002 | 1 | 0.25 |
| C-MRSA10 | Slippers of patient C | 16 | 0.25 | 0.13 | 0.06 | 0.5 | 0.5 | 1 | 2 | 1 | 0.03/0.59 | | 0.5 | 0.002 | 1 | 0.25 |
| C-MRSA11 | Hands of patient C’s son | 16 | 0.25 | 0.13 | 0.06 | 0.5 | 0.5 | 1 | 2 | 2 | 0.03/0.59 | | 0.5 | 0.002 | >256 | 0.13 |
| C-MRSA12 | Hands of patient C’s son | 16 | 0.25 | 0.13 | 0.06 | 0.5 | 0.5 | 1 | 2 | 2 | 0.03/0.59 | | 0.5 | 0.002 | 1 | 0.13 |
| C-MRSA13 | Hands of patient C’s son | 16 | 0.25 | 0.13 | 0.06 | 0.5 | 0.5 | 1 | 2 | 2 | 0.03/0.59 | | 0.5 | 0.002 | 1 | 0.25 |
| C-MRSA14 | Nose of patient C’s son | 16 | 0.25 | 0.13 | 0.03 | 0.5 | 0.5 | 1 | 2 | 2 | 0.03/0.59 | | 0.5 | 0.002 | >256 | 0.25 |
| C-MRSA15 | Nose of patient C’s son | 16 | 0.25 | 0.13 | 0.03 | 0.5 | 0.5 | 1 | 2 | 2 | 0.03/0.59 | | 0.5 | 0.002 | >256 | 0.25 |
| C-MRSA16 | Nose of patient C’s son | 16 | 0.25 | 0.13 | 0.03 | 0.5 | 0.5 | 1 | 2 | 2 | 0.03/0.59 | | 0.5 | 0.002 | >256 | 0.25 |
| D-MRSA1 | Patient D | 8 | 2 | 2 | 1 | 0.5 | 0.5 | 1 | 1 | 4 | 0.03/0.59 | | 0.25 | 0.002 | 1 | 0.25 |
| D-MRSA2 | Television remote control | 32 | 2 | 2 | 1 | 0.5 | 0.5 | 1 | 1 | >128 | 0.03/0.59 | | 0.5 | 0.002 | >256 | 64 |
| D-MRSA3 | Television remote control | 16 | 2 | 2 | 1 | 0.5 | 0.5 | 0.5 | 1 | 1 | 0.03/0.59 | | 0.25 | 0.002 | >256 | 64 |
| D-MRSA4 | Television remote control | 16 | 2 | 1 | 1 | 0.5 | 0.5 | 1 | 1 | 1 | 0.03/0.59 | | 0.25 | 0.002 | >256 | 64 |
| D-MSSA1 | Nose of patient D’s son | 16 | 0.5 | 0.5 | 0.5 | 0.5 | 32 | 0.5 | 1 | 8 | 0.03/0.59 | | 0.25 | 0.002 | >256 | 0.13 |
| E-MRSA1 | Patient E | 8 | 0.5 | 0.5 | 0.5 | 0.5 | 1 | 1 | 1 | 1 | 0.25/4.75 | | 0.25 | 0.002 | 1 | 0.25 |
| H-MSSA1 | H Family male owner's hand | 4 | 2 | 0.5 | 0.5 | 0.5 | 1 | 1 | 1 | 8 | 0.03/0.59 | | 0.5 | 0.002 | 1 | 0.13 |
| J-MSSA1 | Plush toy | 4 | 0.5 | 0.25 | 0.06 | 0.5 | 32 | 1 | 1 | 4 | 0.03/0.59 | | 0.5 | 0.002 | 1 | 0.25 |
| J-MSSA2 | Cat-C | 4 | 0.5 | 0.25 | 0.06 | 0.5 | 32 | 1 | 1 | 4 | 0.03/0.59 | | 0.5 | 0.002 | 1 | 0.25 |
| J-MSSA3 | Computer keyboard and mouse | 4 | 0.5 | 0.25 | 0.06 | 1 | 32 | 1 | 1 | 4 | 0.03/0.59 | | 0.5 | 0.002 | 2 | 0.25 |
| J-MSSA4 | Door handle | 2 | 0.5 | 0.25 | 0.06 | 0.5 | 32 | 0.5 | 1 | 1 | 0.03/0.59 | | 0.5 | 0.002 | 1 | 0.25 |
| J-MSSA5 | Sofa | 4 | 0.5 | 0.25 | 0.06 | 0.5 | 32 | 1 | 1 | 2 | 0.03/0.59 | | 0.5 | 0.002 | 1 | 0.25 |
| J-MSSA6 | J Family male owner's hands | 4 | 0.5 | 0.25 | 0.06 | 0.5 | 64 | 1 | 1 | 1 | 0.03/0.59 | | 0.5 | 0.002 | 1 | 0.13 |
| J-MSSA7 | J Family female owner's nasal | 4 | 0.5 | 0.25 | 0.06 | 2 | 64 | 1 | 1 | 2 | 0.03/0.59 | | 0.5 | 0.002 | 32 | 0.13 |
| J-MSSA8 | J Family male owner's nasal | 4 | 0.5 | 0.25 | 0.06 | 0.5 | 32 | 1 | 1 | 1 | 0.03/0.59 | | 0.5 | 0.002 | 1 | 0.13 |

Abbreviations: FOX, cefoxitin; CIP, ciprofloxacin; LEV, levofloxacin; MXF, moxifloxacin; GEN, gentamycin; TET, tetracycline; LNZ, linezolid; VAN, vancomycin; FOS, fosfomycin; SMZ, trimethoprim-sulfamethoxazole; DAP, daptomycin; RIF, rifampicin; ERY, erythromycin; CLI, clindamycin

## Supplementary Table 2. The SCC*mec* typing of MRSA and MSSA strains sampled from patients, health subjects, and their households.

|  | Specimen source | SCC*mec* typing |
| --- | --- | --- |
| A-MRSA1 | Patient A | V |
| A-MRSA2 | Bathroom sink faucet | V |
| A-MRSA3 | Bathroom sink faucet | V |
| A-MRSA4 | Bathroom sink faucet | V |
| A-MRSA5 | Cat-A | V |
| A-MRSA6 | Cat-A | V |
| A-MRSA7 | Cat-A | V |
| A-MRSA8 | Cat-B | V |
| A-MRSA9 | Sofa | V |
| A-MRSA10 | Sofa | V |
| A-MRSA11 | Hands of patient A’s girlfriend | V |
| A-MRSA12 | Hands of patient A’s girlfriend | V |
| A-MRSA13 | Hands of patient A’s girlfriend | V |
| B-MRSA1 | Patient B | V |
| B-MRSA2 | Elevator button | IV |
| B-MRSA3 | Elevator button | IV |
| C-MRSA1 | Patient C | IV |
| C-MRSA2 | Bathroom sink faucet | IV |
| C-MRSA3 | Bathroom sink faucet | IV |
| C-MRSA4 | Bathroom sink faucet | IV |
| C-MRSA5 | Kitchen sink faucet | IV |
| C-MRSA6 | Kitchen sink faucet | IV |
| C-MRSA7 | Kitchen sink faucet | IV |
| C-MRSA8 | Slippers of patient C | IV |
| C-MRSA9 | Slippers of patient C | IV |
| C-MRSA10 | Slippers of patient C | IV |
| C-MRSA11 | Hands of patient C’s son | IV |
| C-MRSA12 | Hands of patient C’s son | IV |
| C-MRSA13 | Hands of patient C’s son | IV |
| C-MRSA14 | Nose of patient C’s son | IV |
| C-MRSA15 | Nose of patient C’s son | IV |
| C-MRSA16 | Nose of patient C’s son | IV |
| D-MRSA1 | Patient D | V |
| D-MRSA2 | Television remote control | IV |
| D-MRSA3 | Television remote control | IV |
| D-MRSA4 | Television remote control | IV |
| E-MRSA1 | Patient E | V |
